# Supplementary material for: Global Health Priority Box: Discovering Flucofuron as a Promising Antikinetoplastid Compound
Source: Pharmaceuticals (Basel). 2024 Apr 25;17(5):554. doi: 10.3390/ph17050554 (PMC11123942; doi:10.3390/ph17050554)
Supplement: Supplementary file 1 [file pharmaceuticals-17-00554-s001.zip › pharmaceuticals-2890213-supplementary.pdf]

Global Health Priority Box: Discovering Flucofuron as a Promising Antikinetoplastid Compound

Highlights

- New theraphies against leishmaniasis and Chagas disease are needed.
- Compound libraries are a good source of new compounds to test.
- Flucofuron demonstrated activity against *T. cruzi* and *L. amazonensis.*
- Flucofuron produce Programmed Cell Death in *T. cruzi* and *L. amazonensis.*

Supplementary Materials

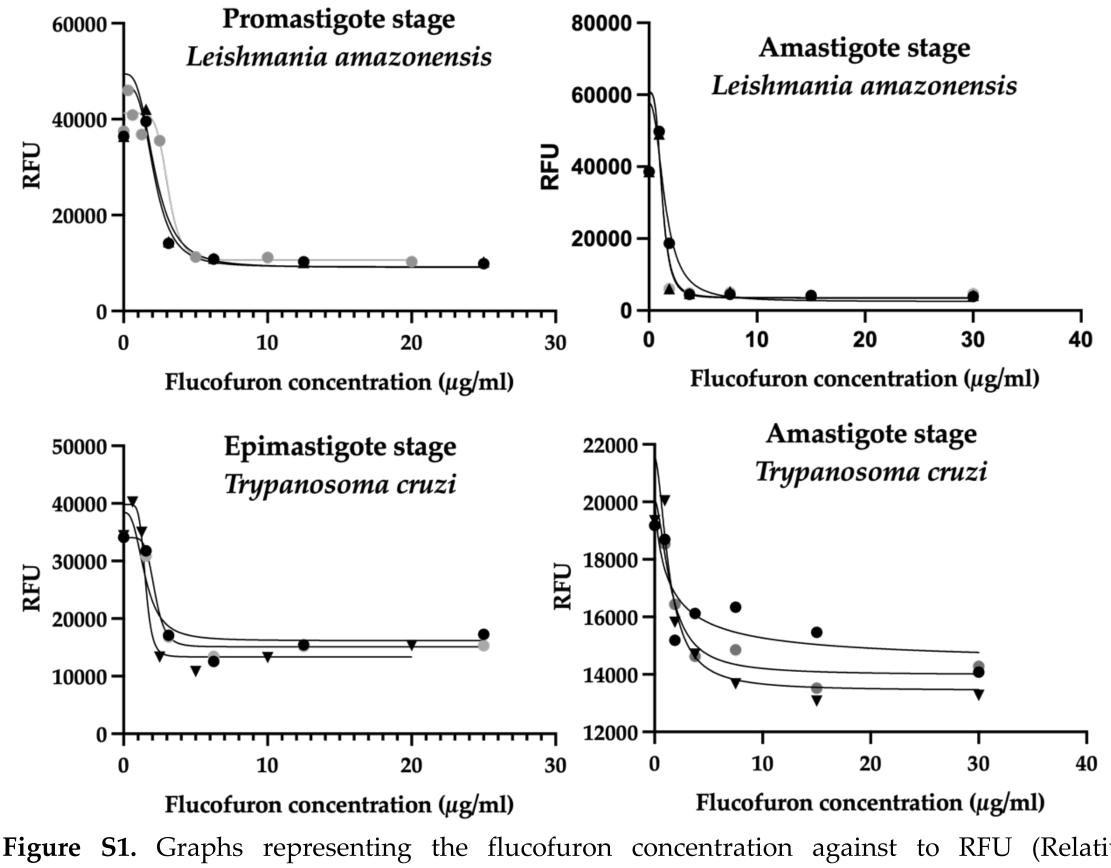

Figure S1. Graphs representing the flucofuron concentration against to RFU (Relative fluorescence units) in the both stages of *Trypanosoma cruzi* and *Leishmania amazonensis*. Each curve represents the two repetitions performed in one day using non-linear regression [Inhibitor] vs response – Variable slope (four parameters) by non-linear regression analysis with 95 % confidence, using the GraphPad Prism 9.0.0 statistical software.
